# Phenotype based clustering, and diversity of common bean genotypes in seed iron concentration and cooking time

**DOI:** 10.1371/journal.pone.0284976

**Published:** 2023-05-11

**Authors:** Winnyfred Amongi, Stanley Tamusange Nkalubo, Mildred Ochwo-Ssemakula, Arfang Badji, Isaac Onziga Dramadri, Thomas Lapaka Odongo, Ephraim Nuwamanya, Phineas Tukamuhabwe, Paulo Izquierdo, Karen Cichy, James Kelly, Clare Mukankusi

**Affiliations:** 1 Department of Agricultural Production, College of Agricultural and Environmental Sciences, Makerere University, Kampala, Uganda; 2 Alliance of Bioversity and CIAT, National Agricultural Research Laboratories Kawanda, Kampala, Uganda; 3 National Crops Resources Research Institute (NaCRRI-NARO), Kampala, Uganda; 4 Makerere University Regional Center for Crop Improvement (MaRCCI), Collage of Agriculture and Environmental Sciences, Makerere University, Kampala, Uganda; 5 Department of Plant, Soil, and Microbial Sciences, Michigan State University, East Lansing, Michigan, United States of America; 6 USDA-ARS, Sugarbeet and Bean Research Unit, East Lansing, Michigan, United States of America; McGill University, CANADA

## Abstract

Common bean is the world’s most important directly consumed legume food crop that is popular for calories, protein and micronutrients. It is a staple food in sub-Saharan Africa, and a significant source of iron for anemic people. However, several pests, soil and weather challenges still impede its production. Long cooking time, and high phytic acid and polyphenols that influence bioavailable iron also limit the health benefits. To inform population improvement strategies and selection decisions for resilient fast cooking and iron biofortified beans, the study determined diversity and population structure within 427 breeding lines, varieties, or landraces mostly from Alliance Uganda and Columbia. The genotypes were evaluated for days to flowering and physiological maturity, yield, seed iron (FESEED) and zinc (ZNSEED) and cooking time (COOKT). Data for all traits showed significant (P≤0.001) differences among the genotypes. Repeatability was moderate to high for most traits. Performance ranged from 52 to 87 ppm (FESEED), 23–38 ppm (ZNSEED), 36–361 minutes (COOKT), and 397–1299 kg/ha (yield). Minimal differences existed between the gene pools in the mean performance except in yield, where Mesoamerican beans were better by 117 kg/ha. The genotypes exhibited high genetic diversity and thus have a high potential for use in plant breeding. Improvement of FESEED and ZNSEED, COOKT and yield performance within some markets such as red and small white beans is possible. Hybridization across market classes especially for yellow beans is essential but this could be avoided by adding other elite lines to the population. Superior yielding and fast cooking, yellow and large white beans were specifically lacking. Adding Fe dense elite lines to the population is also recommended. The population was clustered into three groups that could be considered for specific breeding targets based on trait correlations.

## Introduction

Common bean (*Phaseolus vulgaris* L.) is an important grain legume crop of global importance, especially for human consumption and biological nitrogen fixation. The crop provides calories, protein, and micronutrients, notably iron (Fe), zinc (Zn), thiamin, and folic acid [1]. The annual global production of dry beans in 2019 was approximately 28.9 million tons, with about 7.0 and 4.8 million tons produced in Africa and East Africa, respectively [2]. Over 400 million people in Africa directly consume beans [3]. However, because of several biotic and abiotic production challenges, farmer yields in most African countries that range from 394 to 1589 kg/ha is still below the potential yield of 1500–2000 kg/ha of bush beans [4, 5]. Challenges such as long cooking time, and bioactive compounds such as phytic acid and polyphenols that influence the bioavailability of Fe also affect the consumption and health benefits [6–8].

Estimated average requirement (EAR) of bioavailable iron (micrograms per day) for children aged 6–7 years and women of reproductive age is 500 and 1,460 to which 68 and 42% respectively was estimated to be supplied through the base line content of 50 ppm [9]. A breeding target increment of 44 ppm above the baseline was estimated to supply additional 59 and 38% from a daily consumption of 107 and 198 g of beans (dry weights) by each group, respectively [9]. Thus, a daily consumption of iron-rich beans with the full target level of 94 ppm of Fe was estimated to provide 127% and 80% daily average requirements of children aged 6–7 years and women of reproductive age, respectively [9]. However, not many such genotypes exist on the market despite the realization of 94 ppm through breeding [9, 10]. Increasing the diversity of iron concentration in different market classes, and targeted breeding could increase availability and access to full target level iron-rich beans. It is worth noting that genotype by environment interaction (GxE) was reported as a factor influencing iron concentration in a variety [11]. A recent study indicated that seed iron concentration was largely controlled by genotype, location, and the interaction between genotype, location, and season which represented 25.7, 17.4, and 13.7% of phenotypic variation, respectively [12]. Defining the target population of environments such as sets of farms in which the varieties produced by a breeding program would be grown could lessen GxE effects on iron.

The above strategy is focused on high seed concentration. However, the understanding of factors influencing bioavailable bean Fe remains central to the role of common bean in lessening the burden of anemia in the most vulnerable groups. This would translate to new dietary recommendations especially for groups with limited access to vitamin C (ascorbic acid), if high Fe concentration does not directly equate to high bioavailable Fe. Major inhibitors of bioavailable bean Fe are phytate and polyphenols [13, 14]. The phytate to Fe molar ratios show nearly or maximal inhibition of Fe unless beans are consumed as a composite meal that include enhancers such as ascorbic acid [13, 14]. Soaking beans before boiling caused a significant decrease in total phytic acid and it was recommended as routine practice for increased bioavailable Fe, in addition to breeding for low phytic acid [7, 13]. However, cooking, and presoaking beans were reported to have minimal effect on the net inhibitory role of phytate and the net inhibitory or promoting role of polyphenols on iron bioavailability [8, 15, 16]. Polyphenols, which greatly vary among market classes of beans, was pointed as a major factor contributing to high bioavailable Fe in some market classes such as yellow beans [8, 15, 16]. Efforts are being directed to profiling bean polyphenols to understand which are enhancers and inhibitors of bioavailable iron [8].

Cooking soaked or unsoaked regularly consumed dry beans takes over 1 to 3 hours [6]. This is not only time-consuming, but also requires more fuel and water. These inconveniences reduce per capita consumption of beans hence limiting the health benefits, especially of iron, to the anemia-prone population. Fast cooking beans were associated with more bioavailable Fe [8], which make it an essential trait to consider during biofortification of common bean.

Genetic variation is essential for breeding to result in progressive genetic improvement. A diverse population is a source of desirable genetic variants for breeding, and it is also important as a reference for predicting models for genomic selection [17]. However, such populations tend to be highly structured, which can influence prediction accuracy, heritability, and trait association [18].

Hybridization scheme involving elite x elite genotypes is a key strategy for improving quantitative traits in a short time [19]. To maintain diversity for a long time, new sources of alleles should be regularly introduced to the breeding programme [19]. Nonetheless, recent simulations show that an elite line hybridization scheme can maintain diversity for a long time when an optimized mating strategy is used [20]. When there is inadequate genetic variation for a specific trait among the elite germplasm or within a market class, wide crosses to exotic germplasm are inevitable [21]. These wide crosses could be extended to wild species if trait variation is lacking in *P*. *vulgaris* [21].

Cultivated beans are from two distinct gene pools, the Mesoamerican, and the Andean pools. The Mesoamerican gene pool is characterized by small to medium size seeded beans that weight about 20.0 g to 30.0 g for 100 seeds [22]. The weight of 100 seeds for Andean beans ranges from 35.0 to 50.0 g [22]. Andean and Mesoamerican beans are grown at varying percentages across African countries and regions of the world [23].

The market for common bean is mainly characterized by size and colour attributes but with varying preferences of more than one market class within a country [24]. The popular market classes in Africa include large red mottled (calima), small and large white, yellow, sugar (speckled), dark red kidney, small red, carioca (small stripped), and pinto [24]. In East Africa, the mottled and plain red beans account for approximately 43% of the production, followed by sugar, yellow and white beans at 18, 13, and 9%, respectively [25].

This study aimed to evaluate the genetic diversity and structure of breeding lines and varieties to inform population improvement strategies and selection decisions for resilient fast cooking and Fe biofortified beans.

## Materials and methods

### Plant material

The genotypes consisted of 427 bush bean breeding lines that were developed from parents with resistance to common bacterial blight (CBB) and angular leaf spot (ALS), drought tolerance, fasting cooking time and seed iron/zinc concentration (Table 1).

**Table 1:** The key traits and background of genotypes.

Genotypes	Trait	Background
KNG1-KNG12	Earliness to cook, iron, zinc	AWASH1, Ngwakungwaku, Awash Melka, VAX4, ACC714, CAL96, NABE3, KATX56b
KNG13-KNG31	Drought	BFS27, SCR9, NCB226, CAL96, NABE15, MASINDI YELLOW LONG, MALAWI GREEN, NCB226, BFS27
KNG32-KNG34	ALS^a^	CAL96, MEXICO54, AND277, G5686, Kanyebwa, U000297
KNG35-KNG50	Iron, zinc, drought	BFS10, ALB6, NUAK576, NUAK512, SAB713, CAB2, SMC28, Malawi Green, KATB1, DAB366, RWR2154, NUAK399, DAB302, DAB441, NUAK515
KNG51-KNG532	CBB^b^, iron, zinc	MIB 456, MCM 2001, RWR 2154, RWV 2001, Jesca, NUA 45, Kanyebwa, Masindi Yellow, K131, CAL96

^a^Common bacterial blight.

^b^Angular leaf spot.

The genotypes are of diverse backgrounds including small, medium and large seeded beans that fall into two of the three common bean breeding pipe lines namely Andean and Mesoamerican bush beans.. The Andean (medium-large seeded bush beans) and Mesoamerican (small seeded bush beans) pipelines represent 74 and 10% of market in Africa, respectively. The climber pipeline that represents 16% of the market in Africa was not included in this study. The seven major market segments in the two pipelines include Andean bush red mottled, Andean bush red, Andean bush cream striped, Andean bush yellow, Meso bush red, Meso bush white and Meso bush black. In consideration of all the market segments, the major common bean market classes in Africa, including red and red mottled (Calima), sugar (speckled), white and yellow beans, consisted of 40.4% of all the genotypes (Table 2).

**Table 2:** Market class based on seed colours within gene pools of 472 common bean genotypes.

	Andean	Mesoamerican	Admixture
Total	87	305	35
Black	0	43	1
Brown	3	6	2
Cream (Pinto, Carioca, Mulatinho)	2	159	5
Others	9	16	18
Pink	1	0	0
Purple	5	0	1
Red	7	51	2
Red Mottled	15	0	0
Sugar (Speckled)	23	1	3
White	9	26	3
Yellow	13	3	0

### Trial location and establishment

The genotypes were evaluated at the National Agricultural Research Laboratories (NARL)-Kawanda and Kitengule prison farm for two seasons, A and B, in 2018 (A = March-June and B = September-December). Further evaluation was performed at the NARL-Kawanda and Rwebitaba zonal agricultural research and development institute (ZARDI) in the first season (A) of 2020. The NARL-Kawanda is located at 32°31’E, 0°25′N at an altitude of 1,190 m above sea level (asl) in central Uganda; Kitengule is located at 2°08’S, 33°26’E at an elevation of 1,320 m asl in western Tanzania. Rwebitaba ZARDI is located at 0.6932°N, 30.3330°E at an altitude 1,506 m asl in western Uganda. The trials were established in an alpha lattice design with two replications. Plots of three rows that were 3 m long, and with row and plant spacing of 50 cm and 10 cm were used. Hand application of granular NPK 17:17:17 fertilizer was performed at the rate of 125 kg/ha just before planting. Each trial was weeded twice, and an insecticide (Dimethoate) and two fungicides (Mancozeb and Ridomil) were applied using the manufacturers’ rates.

### Data collection

Data were collected on days to 50% flowering (DF) and physiological maturity (DPM), yield (YDHA), seed iron (FESEED), zinc (ZNSEED) concentration, and cooking time (COOKT). The DF and DPM were recorded as number of days from planting to the day when 50% of plants had at least one flower, and number of days from planting to the day when the first pods began to discolor in 50% of the plants, respectively [26, 27]. Seed harvesting for yield data began when 90% of the pods discoloured. The seeds were sun-dried to less than 13% moisture content and sorted for foreign matter before recording total seed weight per plot.

### Cooking time analysis

Twenty-five damage-free seeds that were less than three-months-old, and of moisture contents 10–13%, were randomly sampled from each plot, weighed (g) and soaked in distilled water at room temperature (22 ± 2 ◦C) for 18 hours, drained, re-weighed and then kept in sealed bottles till the initiation of the cooking process [28, 29]. Hydration coefficient (HC), which measures the soaking ability, was calculated as: $\frac{Weight\;of\;soaked\;beans\;(g)}{Dry\;bean\;weight\;(g)}$. Seeds from each plot were positioned into each of the 25 holes of the Matson cooker so that the piercing tips of the 90 g rods were in contact with the surface of the bean seeds prior to placing them in a five-litter beaker containing distilled boiling water [28]. The optimum cooking time was defined as the time required for 80% of the plungers to penetrate the seeds [28].

### Iron and zinc analysis

Well-filled 10–15 pods hanging above the soil were randomly sampled per plot and placed in clean envelopes before the main harvest, hand threshed, wiped with distilled water to remove any soil contamination before packing the seeds in clean paper bags. Each sample was oven dried in paper bags at 60 ºC for 60 hours and ground for 5 minutes to flour using a Retsch Mixer Mill MM 400 fitted with ZrO grinding jars and balls (Retsch GmbH & Co KG, Haan, Germany). The Jars were washed using soap and distilled water and blotted dry using paper towel between samples. Flour samples were analyzed using the ThermoFisher Scientific ARL QUANT’X Energy Dispersive X-ray Fluorescence (EDXRF) model, at NARL, Kawanda. Samples were prepared and analysed using the procedures described in the user manual for ARL QUANT’X [30]. In summary, sample cups carefully fitted with XRF films were halfway filled, and slightly pressed to level-up and fill the cup base. Before loading samples to the machine, energy adjustment was performed using a standard copper sample to verify whether the parameter values of Final Fine Gain Setting meet the specification of between 25000–30000 and Peak FWHM (eV) between 150-170eV. Ten samples per run were loaded to specified positions, and the amount of Fe and Zn were determined by scanning each sample for 60 seconds per element, and then spinning the sample cups to analyze all samples and record the intensities of emitted X-rays. The results of the analysis were displayed automatically when all samples on the tray were measured. Standards with known Fe concentrations are routinely run as quality check for the analysis of Fe and Zn.

### Data analysis

Single environment data was analyzed using breeding view software v1.8.0.52 that uses ASREML [31] to assess for within-trial variability. Cleaned trial data was then subjected to a combined analysis of variance (ANOVA) in META-R v6.0 using the linear model: *Y*_*ijkl*_ = *GM* + *E*_*i*_ + *R*_*j*_(*E*_*i*_) + *B*_*k*_(*E*_*i*_*R*_*j*_) + *G*_*l*_ + *G*_*l*_*E*_*i*_ + ε_*ijkl*_. Y_ijkl_ described the observed value, GM the Grand Mean, E_i_ the Environment effect, R_i_ the Replication effect, B_K_ the Block effect, G_l_ the genotype effect, GE_li_ the Genotype x Environment effect, and ε_ijkl_ the error [32]. All the effects were considered random, and repeatability (broad-sense heritability, H^2^) was calculated as: $\frac{{VC}_{G}}{{VC}_{G}+{{VC}_{GE/nE}+VC\varepsilon }_{/nRnE}}$, where VC described the Variance Components, G the Genotype, GE the Genotype by Environment, n the numbers, E the Environment, ε the error and R the Replication effects [32]. Cluster and principal component analyses were performed in R software v4.1.2 using means from best linear unbiased prediction (BLUP). The phenotype data were standardized, and hierarchical clustering based on Euclidian matrix distance was performed through the NbClust package using the wardD2 method [33]. The PCA was performed by singular value decomposition [34] and the first two components were plotted to visualize relationships among genotypes. Overall breeding values were used to estimate genetic correlations among traits. Desire software (https://bkinghor.une.edu.au/desire.htm) was used to define the weights and build the optimized index. Heritability (repeatability), genetic correlation matrix, scaled standard deviations and economic indices for traits were imported to desire and used to assign weights. Weights were assigned as -0.07 (DF), -0.07 (DPM), 0.37 (YDHA), -0.11 (COOKT), 0.05 (HC), 0.16 (FESEED), 0.19 (ZNSEED) which represented 40.5% economic response based on original economic weights for the traits (which was left as default (one)).

## Results

### Diversity of the genotypes

#### Analysis of variance

A combined analysis of variance showed that repeatability was moderate to high for most traits ranging from 0.32 in yield to 0.81 in Zn (Table 3). Genotypes were significantly different (P≤0.001) in all traits, which indicated diversity. The interaction of genotypes with the environment were also significant (P≤0.001).

**Table 3:** Analysis of variance (ANOVA) for the evaluated phenotypes of 427 common bean genotypes.

Statistic	DF^a^	DPM^b^	YDHA^c^	COOKT^d^	HC^e^	FESEED^f^	ZNSEED^g^
Repeatability/ Heritability	0.80	0.69	0.32	0.65	0.45	0.68	0.81
Genotype Variance	3.5***	2.0***	5515***	343.2***	0.01***	15.8***	5.4***
Gen^h^xEnvt Variance	2.8***	1.6***	21247***			21.3***	3.0***
Envt^i^ Variance	23.0***	16.8*	32333*			42.4***	28.6***
Residual Variance	3.0	5.9	75938	362.2	0.02	48.3	9.1
Grand Mean	44.1	75.2	791.0	73.0	1.9	64.2	28.9
Range	36–49	69–80	397–1299	36–361	1.6–2.1	52–87	23–38
LSD^j^ (P = 0.05)	2.3	2.2	172	26	0.2	6.4	2.9
SD^k^	1.7	2.4	275.6	19.0	0.1	6.9	3.0
CV^l^ (%)	3.9	3.2	34.8	26.1	8.17	10.8	10.4
n^m^ Replicates	2	2	2	2	2	2	2
n Environments	5	5	5	1	1	6	6

^a^Days to 50% flowering.

^b^Days to 50% physiological maturity.

^c^Yield (kg/ ha).

^d^Cooking time (minutes).

^e^Hydration coefficient.

^f^Seed iron concentration (ppm, parts per million).

^g^Seed zinc concentration (ppm).

^h^Genotype.

^i^Environment.

^j^Least significant difference.

^k^Standard deviation.

^l^Coefficient of variation.

^m^Number.

#### Days to flowering (DF), maturity (DPM), and yield performance

**Fig 1:**
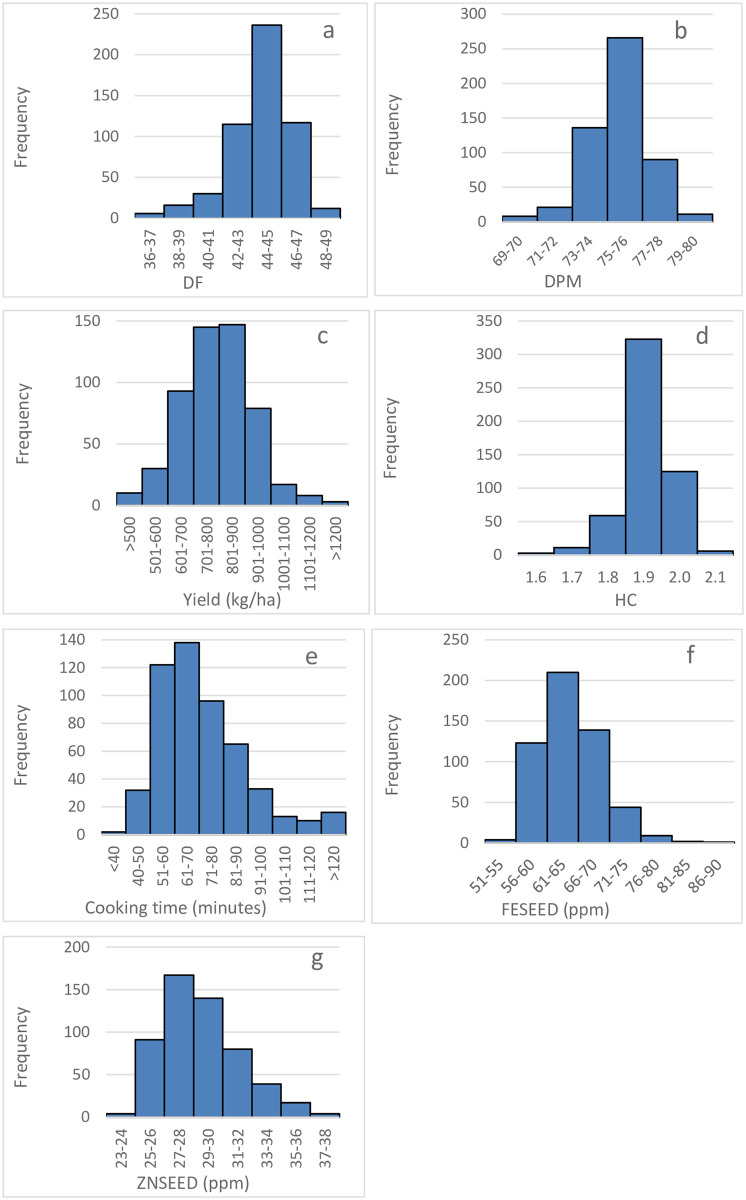
Days to 50% flowering (DF) and maturity (DPM), yield, hydration coefficient (HC), cook time (COOKT), seed iron (FESEED) and zinc (ZNSEED) concentration of 427 common bean genotypes evaluated in multiple environments.

**Fig 2:**
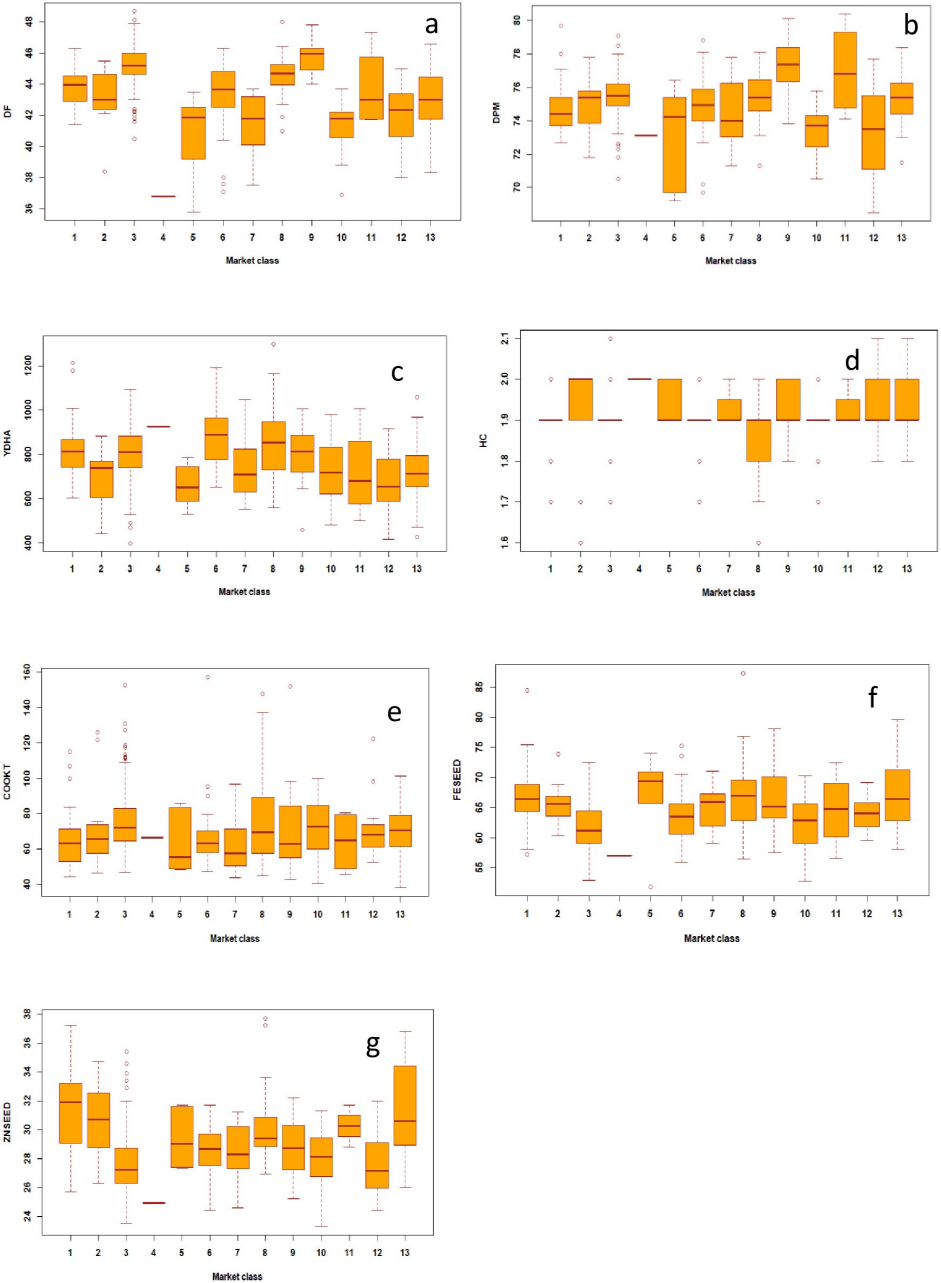
Box plots for days to 50% flowering (DF) and maturity (DPM), yield, hydration coefficient (HC), cook time (COOKT), seed iron (FESEED) and zinc (ZNSEED) concentration of 427 common bean genotypes within market class, evaluated in multiple environments. Note: 1 = Black market class, 2 = Brown market class, 3 = Cream market class, 4 = Pink market class, 5 = Purple market class, 6 = Red market class (medium–large), 7 = Red mottled market class, 8 = Small red market class, 9 = Small white market class, 10 = Sugar (Speckled) market class, 11 = White market class (medium–large), 12 = Yellow market class, 13 = Others. A box plot indicates the position of the minimum, maximum and median values along with the position of the lower and upper quartiles.

**Fig 3:**
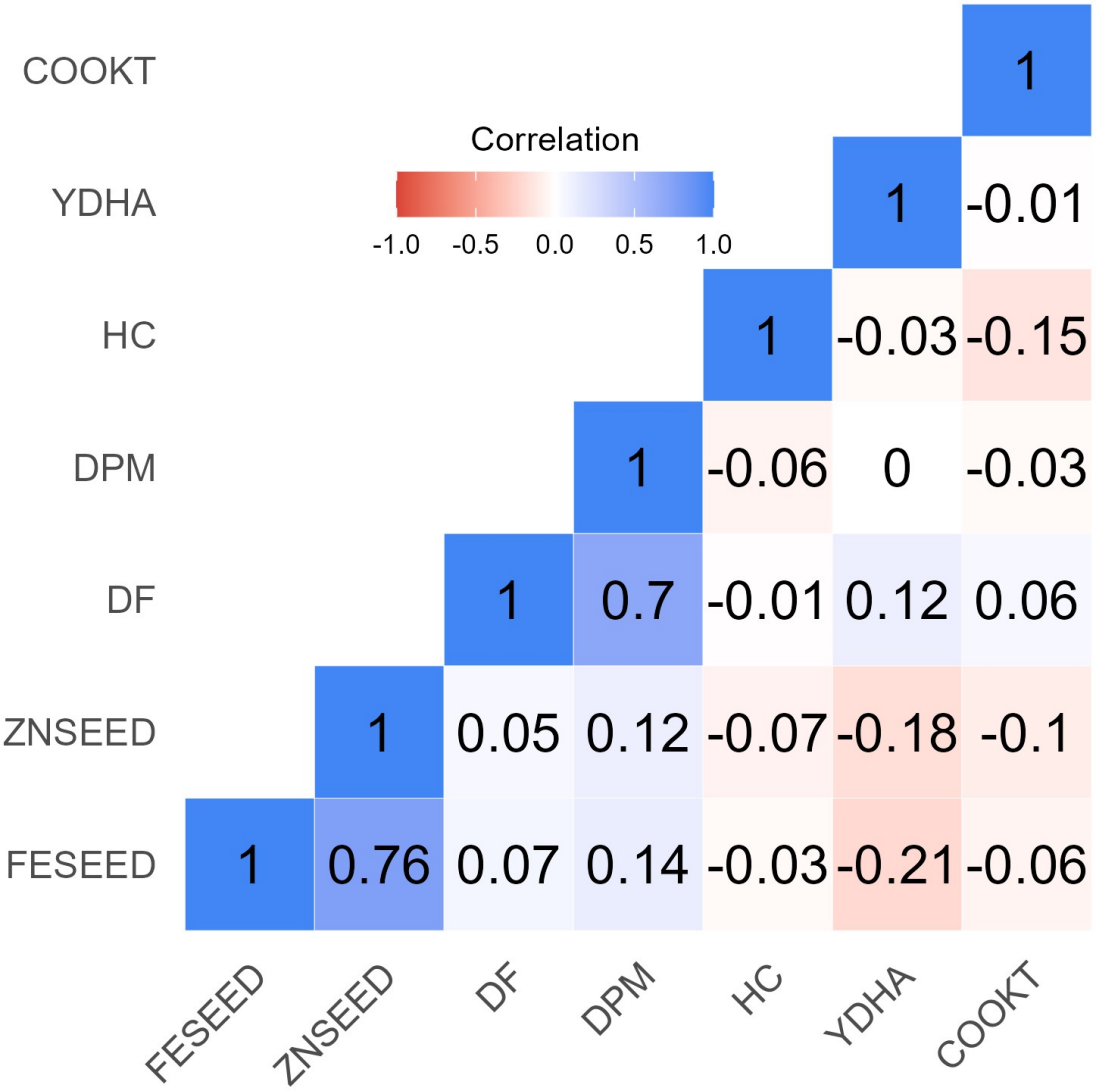
Estimate genetic correlations among traits of 427 common bean genotypes. Days to 50% flowering (DF) and maturity (DPM), yield, cook time (COOKT), hydration coefficient (HC), seed iron (FESEED) and zinc (ZNSEED) concentration.

**Fig 4:**
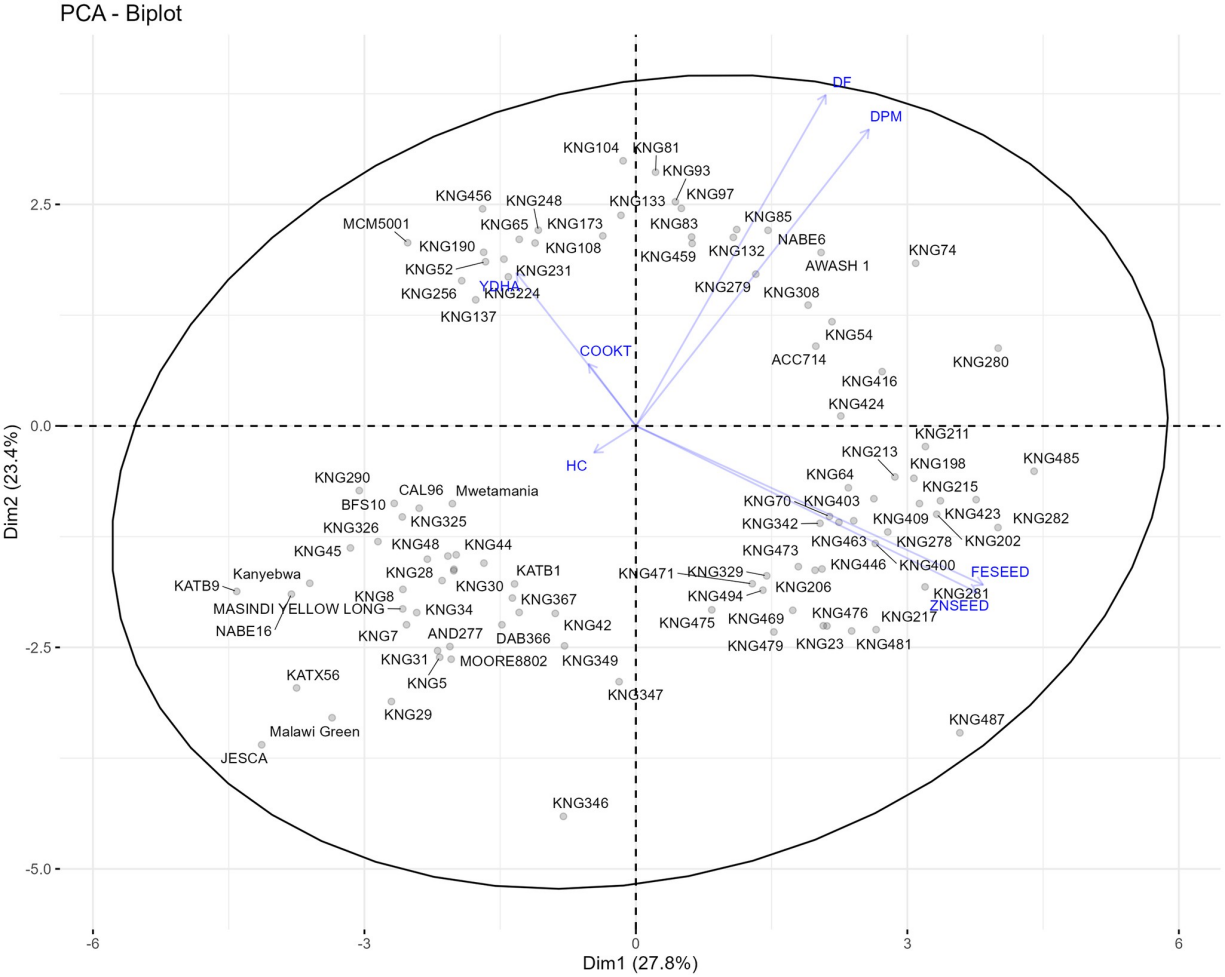
Principal component analysis biplot showing best genotypes for each trait of 427 common bean genotypes. Days to 50% flowering (DF) and maturity (DPM), yield, cook time (COOKT), hydration coefficient (HC), seed iron (FESEED) and zinc (ZNSEED) concentration.

**Fig 5:**
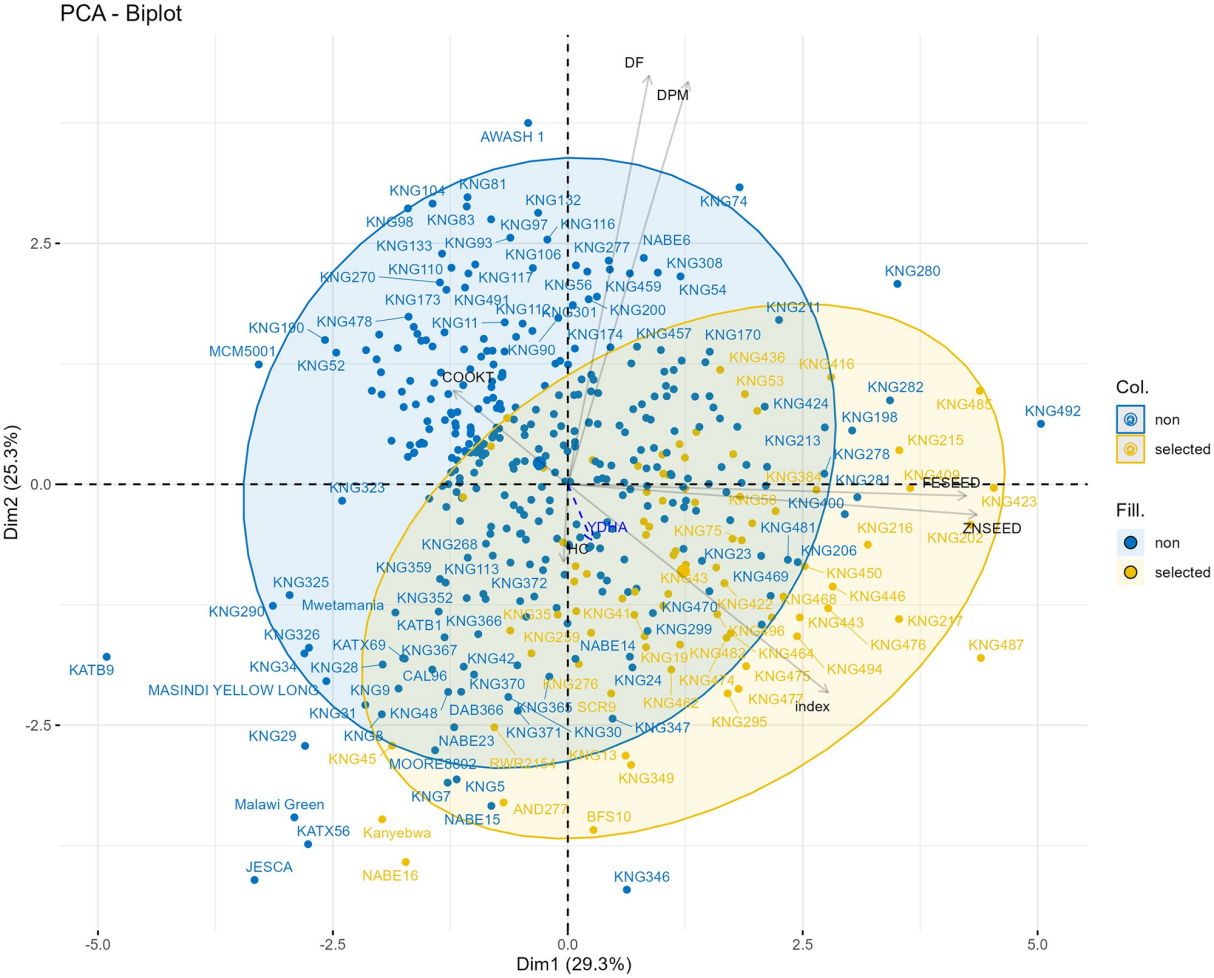
Principal component analysis biplot showing best genotypes for each trait and selected or non-selected genotypes based an optimized selection index. Days to 50% flowering (DF) and maturity (DPM), yield, cook time (COOKT), hydration coefficient (HC), seed iron (FESEED) and zinc (ZNSEED) concentration.

The variations ranged from 36 to 49 and 69 to 80 for DF (Fig 1a) and DPM (Fig 1b), with means of 45 and 75 (Table 3), respectively. This reflected the possibility of breeding for genotypes that flower and mature early. Overall, majority of purple beans flowered and matured early while more than half of the small to large white beans matured late (Fig 2a and 2b). DF and DPM had a strong positive correlation of 0.70 (Fig 3). The earliest genotypes to flower and mature were JESCA at 36 days and KNG29 at 69 days, respectively (Fig 4 and Table 4). Index selection included several early flowering and maturing genotypes but JESCA and KNG29 were excluded (Fig 5).

**Table 4:** Best Linear Unbiased Estimator (BLUE, mean) of performance across or within environment (s), selection index, gene pool, genotype-based clusters, market class and release status of selected common bean genotypes.

Genotype	DF^a^	DPM^b^	YDHA^c^	FESEED^d^	ZNSEED^e^	COOKT^f^	HC^g^	Index	Market class	Gene pool	Phenotype based clusters	Release status
	All Environments					One location						
KNG29	40.9	68.5	518.5	62.1	25	69.9	1.9	2.6	Yellow	Andean	2	Breeding line
KNG30	41.6	71.4	777.2	66.4	26.8	64.2	1.9	1.8	Yellow	Andean	2	Breeding line
KNG372	43.8	73.1	726.4	60.7	28	66.2	2.1	1.09	Yellow	Andean	2	Breeding line
KNG362	43.1	74.3	648	68.1	28.3	52.9	1.9	0.21	Yellow	Andean	2	Breeding line
KNG359	42	73.9	638	62.6	26.2	61.6	2	-0.35	Yellow	Andean	2	Breeding line
KNG354	42.7	75.1	414	64.3	27.5	52.8	1.9	-1.29	Yellow	Andean	2	Breeding line
KNG47	42.7	77.4	812.7	69.2	32	66.3	1.8	-1.46	Yellow	Mesoamerican	1	Breeding line
SMC28	45	77.7	894.1	68.2	31.9	71.5	1.9	-0.75	Yellow	Mesoamerican	1	Breeding line
KATB1	41.3	72.7	625.9	65.3	26	73.5	2	0.23	Yellow	Andean	2	Variety
MOORE8802	39.4	71.3	675.1	61.1	28.6	97.9	2	0.56	Yellow	Andean	2	Variety
MASINDI YELLOW LONG	40.2	70.9	656.9	61.5	24.4	72.3	1.8	0.94	Yellow	Andean	2	Land race
Malawi Green	38	69.7	589	59.5	24.5	56.7	1.9	0.89	YellowGreen	Andean	2	Land race
KNG259	41.7	74.1	1005	56.6	28.8	45.5	1.9	0.41	White	Mesoamerican	2	Breeding line
KNG37	41.8	78.2	649.3	65.8	30.3	52	1.9	-2.91	White	Admixture	3	Breeding line
SAB713	44.2	75.4	501.2	63.7	31.7	77.9	2	-0.64	White	Admixture	1	Variety
KNG276	41.8	72.9	956.8	66.7	27.8	84.6	1.9	1.22	Sugar	Andean	2	Breeding line
KNG299	42.3	73.3	781.3	68	30.4	65.8	1.9	1.04	Sugar	Andean	1	Breeding line
KNG337	42	73.4	797.7	62.9	31.2	40.8	1.9	0.94	Sugar	Andean	2	Breeding line
KNG45	38.8	71.9	977.9	58.5	25.8	92.9	1.8	0.41	Sugar	Andean	2	Breeding line
KNG295	40.7	74.3	963.8	68.2	31.3	65.5	1.9	0.32	Sugar	Andean	2	Breeding line
KNG328	42.4	74.3	767.3	64.2	29.8	48.4	1.9	0.26	Sugar	Andean	1	Breeding line
KNG333	43	74.9	657.1	61	28.1	56.6	2	-0.54	Sugar	Andean	2	Breeding line
KNG341	43.3	75.8	705.7	62.9	30.2	43.9	2	-0.63	Sugar	Andean	1	Breeding line
NABE23	39	73.7	833.6	58.8	26.9	58.9	2	-0.83	Sugar	Andean	2	Variety
DAB366	40.6	71.9	653.2	62.9	27.4	54.2	1.9	0.77	Sugar	Andean	2	Breeding line
RWR2154	41.8	70.5	933.2	64.6	27.2	97.2	1.9	2.63	Sugar	Andean	2	Variety
NABE15	36.9	72.1	622.4	64.6	28.3			NA	Sugar	Andean		Variety
KNG207	44.5	73.8	835.4	61.8	30.1	47.4	2	1.45	Small white	Mesoamerican	1	Breeding line
KNG88	46.2	76.2	970.9	64.2	27.6	84.3	1.9	0.34	Small white	Mesoamerican	3	Breeding line
KNG58	44	76.3	1005.3	70.5	31.3	80.9	1.9	0.15	Small white	Mesoamerican	1	Breeding line
KNG418	44.3	76.4	938.5	69.5	30.2	50.5	2	0.09	Small white	Mesoamerican	1	Breeding line
KNG424	46	77.2	715.8	71.4	31.1	42.7	2	-0.25	Small white	Mesoamerican	1	Breeding line
KNG53	45.8	77.9	974.5	67.8	30.4	58.3	1.9	-0.46	Small white	Mesoamerican	1	Breeding line
KNG416	45.9	78.3	838.7	73	31.6	44.6	1.9	-0.66	Small white	Mesoamerican	1	Breeding line
KNG410	44.1	77.3	707.5	71.2	29.3	45.5	1.9	-1.16	Small white	Mesoamerican	1	Breeding line
Awash Melka	45	77.5	738.1	64.1	25.6	53.5	1.9	-1.53	Small white	Mesoamerican	3	Variety
NABE6	46.1	80.1	844.4	65.2	28.5	61	1.9	-2.5	Small white	Mesoamerican	3	Variety
AWASH 1	47.8	79.9	458.3	63.5	28.6	62.6	1.9	-2.81	Small white	Mesoamerican	3	Variety
KNG383	45.8	75.3	1005.6	68.4	30.5	57.5	1.9	1.49	Small red	Mesoamerican	1	Breeding line
KNG485	48	76.4	708.4	76.8	37.7	78.1	2	1.46	Small red	Mesoamerican	1	Breeding line
KNG381	44.1	75.3	1165.6	67.2	30.2	44.9	2	1.32	Small red	Mesoamerican	1	Breeding line
KNG35	42.7	73.6	1023.7	56.5	28.4	55.7	1.9	1.05	Small red	Admixture	2	Breeding line
KNG458	44.3	75.3	870.9	63	29.3	52.1	2	0.36	Small red	Mesoamerican	1	Breeding line
KNG459	45.6	77.3	1044.3	65	29	124.2	1.6	-0.58	Small red	Mesoamerican	1	Breeding line
KNG120	46	77.3	935.5	60.7	27.5	74.6	1.8	-0.67	Small red	Mesoamerican	3	Breeding line
KNG457	44.3	78.1	956.2	67.4	28.4	89	1.7	-1.54	Small red	Mesoamerican	1	Breeding line
KNG492	45.2	76.6	630	87.3	37.2			NA	Small red	Admixture		Breeding line
BFS10	41	71.3	1299.2	59.1	28.8	62.9	1.9	2.86	Small red	Mesoamerican	2	Breeding line
SCR9	41.9	73.1	1023.1	62	29.6	56	1.9	1.43	Small red	Mesoamerican	2	Breeding line
NABE3	45	75.1	810.6	65.9	28.9	70.3	1.9	0.55	Small red	Mesoamerican	1	Variety
KNG352	42.7	72.6	803.3	61.4	25.8	57.5	1.9	1.18	Red mottled	Andean	2	Breeding line
KNG365	40.7	73	729	67.4	27.2	48.9	1.9	0.41	Red mottled	Andean	2	Breeding line
KNG347	39.4	73.1	641.9	66.7	30.6	49.3	1.9	-0.09	Red mottled	Andean	2	Breeding line
KNG335	43.7	76.3	883.1	64.9	30	51.2	1.9	-0.38	Red mottled	Andean	1	Breeding line
KNG336	43.4	76.5	552.4	67.6	31.2	50.2	1.9	-1.26	Red mottled	Andean	2	Breeding line
KNG343	42.7	76.2	561.6	65.9	30	44	1.9	-1.38	Red mottled	Andean	2	Breeding line
KNG330	43.1	77.8	626.4	66.6	30.4	52.3	1.9	-2.2	Red mottled	Andean	1	Breeding line
CAL96	40.5	73.3	836.9	59.2	25.2	58	1.9	-0.13	Red mottled	Andean	2	Variety
KATX69	39.6	73.1	709	59.2	27.4	88.7	1.9	-0.66	Red mottled	Andean	2	Variety
NABE16	37.5	71.3	1048.1	59.1	24.6	69.7	1.9	0.65	Red mottled	Andean	2	Variety
NABE4	41.8	74	656.6	62.5	27.5	55.7	2	-0.31	Red mottled	Andean	2	Variety
KNG13	41	72.8	1116.2	61.8	29.4	52.1	1.9	1.57	Red	Mesoamerican	2	Breeding line
KNG379	45.7	75.8	1135.2	65.1	29	63.1	1.9	1.15	Red	Mesoamerican	1	Breeding line
KNG393	43.3	74.1	971.8	63.3	29.6	60.5	1.9	1.1	Red	Mesoamerican	2	Breeding line
KNG397	44.9	74.9	849.8	68.8	30.1	52.8	1.9	1.08	Red	Mesoamerican	1	Breeding line
KNG41	42.8	74.6	1192.8	63.8	28	58.3	1.9	1.04	Red	Mesoamerican	2	Breeding line
KNG15	41.9	73.5	969.7	61.7	29.7	67	1.7	0.94	Red	Mesoamerican	3	Breeding line
KNG447	44.4	75	965.6	66.8	28.7	63.3	1.9	0.87	Red	Mesoamerican	1	Breeding line
KNG384	44.9	75.9	933.3	75.2	31.2	79.6	1.9	0.74	Red	Mesoamerican	1	Breeding line
KNG50	42.6	73.9	774.3	60.4	28.6	47.5	1.8	0.4	Red	Mesoamerican	2	Breeding line
KNG36	42.8	75.1	984.7	61.9	28.5	58.5	1.9	0.13	Red	Admixture	2	Breeding line
KNG456	45.3	76.8	1169.6	58	25	76.5	2	-0.28	Red	Mesoamerican	3	Breeding line
KATX56	37.1	70.2	734.9	57	25.5	78	1.9	0.43	Red	Andean	2	Variety
NABE13	42.2	74.3	947.5	56	27.6	57.9	1.9	0.1	Red	Andean	2	Variety
AND277	38	72.7	808.7	64.7	26.3	55	2	-0.32	Red	Andean	2	Variety
KATB9	37.6	69.7	738.3	60.6	24.4	307.3	1.9	-0.53	Red	Andean	1	Variety
NABE14	40.4	74	650	64.8	30	63.6	2	-0.58	Red	Andean	2	Variety
ALB6	42.5	77.4	979.2	65.6	28.1	66.9	2	-1.56	Red	Mesoamerican	1	Variety
KNG346	39.2	69.2	667.3	74.1	28.7	58.4	2	2.72	Purple	Andean	2	Breeding line
KNG358	42.3	73.8	742.9	70.1	29.3	52.3	1.9	0.68	Purple	Andean	1	Breeding line
KNG329	42.5	75.4	530.3	68.7	31.6	48.9	1.9	-0.77	Purple	Andean	2	Breeding line
KNG342	43.5	76.4	588.7	70.9	31.7	48.4	1.9	-0.88	Purple	Admixture	2	Breeding line
JESCA	35.8	69.7	630.8	51.9	27.4	85.6	1.9	-0.03	Purple	Andean	2	Variety
KNG317	44.9	74.2	1048.4	61.4	27.8	60	2	1.56	Pinto	Mesoamerican	3	Breeding line
KNG75	45.8	75.1	998.6	68.1	30.8	71.2	2.1	1.53	Pinto	Mesoamerican	1	Breeding line
KNG193	45	74.2	836.1	65.2	31	49.5	2	1.53	Pinto	Mesoamerican	1	Breeding line
KNG229	44.2	73.6	990.2	59.8	26.4	54.6	1.8	1.47	Pinto	Mesoamerican	3	Breeding line
KNG131	46.5	75.4	938.8	66.2	30.1	63.1	1.9	1.34	Pinto	Mesoamerican	1	Breeding line
KNG91	45.6	74.6	942.8	60.9	27.3	62.6	2	1.18	Pinto	Mesoamerican	3	Breeding line
KNG156	45.2	74.4	938.4	57.2	26.4	60.7	1.9	0.98	Pinto	Mesoamerican	3	Breeding line
KNG177	46.2	75.5	999.4	62.6	26.8	64.7	1.9	0.91	Pinto	Mesoamerican	3	Breeding line
KNG194	46	74.8	940	58.2	27.6	91.9	1.9	0.9	Pinto	Mesoamerican	3	Breeding line
KNG78	45.7	76	981.2	67.7	31.4	81.9	1.9	0.78	Pinto	Mesoamerican	1	Breeding line
KNG231	46.2	75.4	991.5	57.6	25.2	59.1	1.9	0.68	Pinto	Mesoamerican	3	Breeding line
KNG86	45.4	75.3	942.5	63.9	26.5	65.3	1.9	0.66	Pinto	Mesoamerican	3	Breeding line
KNG108	46.1	75.8	1089.9	58.4	27.1	89.9	1.9	0.59	Pinto	Mesoamerican	3	Breeding line
KNG145	45.2	75.5	981.5	65.1	27.8	82.7	1.9	0.57	Pinto	Mesoamerican	3	Breeding line
KNG62	45.7	75.9	998.5	65	30.6	106.2	2	0.56	Pinto	Mesoamerican	1	Breeding line
KNG241	46.2	75.7	973.8	59.2	26.8	72.4	1.9	0.52	Pinto	Mesoamerican	3	Breeding line
KNG266	43.8	75.2	948.4	68	28.1	62.9	1.9	0.49	Pinto	Mesoamerican	1	Breeding line
KNG256	44.6	75.4	1093.6	56.2	25.6	56.6	1.9	0.38	Pinto	Mesoamerican	3	Breeding line
KNG309	46.3	75.3	526.7	68.3	28.9	53.3	1.8	0.35	Pinto	Mesoamerican	1	Breeding line
KNG67	44.7	75.9	993	61.1	27.1	64.1	1.9	0.07	Pinto	Mesoamerican	3	Breeding line
KNG313	44.9	76.5	948.2	66.7	30.7	74.2	1.8	0.03	Pinto	Mesoamerican	1	Breeding line
KNG239	44.7	75.5	961.4	60.2	24.9	70.5	2	0.02	Pinto	Mesoamerican	3	Breeding line
KNG271	44.4	76.2	849.9	69.7	29.5	46.5	1.9	0.02	Pinto	Mesoamerican	1	Breeding line
KNG288	44.7	75.7	822.8	63	27.9	52.7	2	-0.01	Pinto	Mesoamerican	1	Breeding line
KNG224	45.1	75.8	999.2	59.4	25.1	75.4	1.9	-0.01	Pinto	Mesoamerican	3	Breeding line
KNG80	43.8	75.5	954	61.7	28	77.3	1.9	-0.06	Pinto	Mesoamerican	3	Breeding line
KNG242	44.8	76.1	938.8	63.2	27.4	65	1.9	-0.08	Pinto	Mesoamerican	3	Breeding line
KNG150	44.6	76.5	970.8	65.8	28	56.7	1.9	-0.15	Pinto	Mesoamerican	1	Breeding line
KNG180	43.6	75.5	1027.3	58.5	26	90.8	1.9	-0.31	Pinto	Mesoamerican	3	Breeding line
KNG94	44.1	76.8	990.9	66.4	28.7	66.6	1.9	-0.47	Pinto	Mesoamerican	1	Breeding line
KNG251	46.1	76.8	776.5	59.6	27.1	51	1.9	-0.6	Pinto	Mesoamerican	3	Breeding line
Kanyebwa	36.8	73.1	925.3	57	24.9	66.4	2	-1.21	Pink	Andean	2	Land race
KNG356	44.1	73.9	777.3	72.5	29.6	54.8	1.9	1.42	Cream (Mulatinho)	Andean	1	Breeding line
KNG348	42.2	74.6	584.8	67	28.9	52.2	1.9	-0.47	Cream (Mulatinho)	Andean	2	Breeding line
Mwetamania	41.8	73.3	719.5	56.5	27.3	70.1	2	0	Cream (Mulatinho)	Mesoamerican	2	Variety
MCM5001	44.4	76.1	861.5	52.9	23.5	111.6	1.9	-1.42	Cream (Mulatinho)	Mesoamerican	3	Variety
KNG483	43	74.1	442.6	65.6	31.2	46.3	2	-0.03	Brown	Mesoamerican	2	Breeding line
G5686	42.1	75.4	668.8	61.4	26.3	56.2	1.9	-1.3	Brown	Andean	2	Breeding line
KNG19	43.7	73.3	1213.4	60	30	48.1	1.8	2.4	Black	Mesoamerican	2	Breeding line
KNG442	44	73.3	976.9	66.6	31	107	2	1.84	Black	Mesoamerican	1	Breeding line
KNG487	42.5	73.5	640.3	84.5	35.4	58.7	1.9	1.68	Black	Mesoamerican	1	Breeding line
KNG386	44.2	73.6	949.4	62.4	28.2	47.6	1.9	1.65	Black	Mesoamerican	3	Breeding line
KNG464	43.2	73.6	728.7	67.4	33.2	49.4	2	1.31	Black	Mesoamerican	1	Breeding line
KNG496	43	73.9	786.3	66.2	32.9	47.9	1.9	1.12	Black	Mesoamerican	1	Breeding line
KNG396	44.6	75	1179.6	58.1	27.9	63.6	1.9	1.09	Black	Mesoamerican	2	Breeding line
KNG477	41.9	73.7	826.2	66.5	33.2	53.1	2	0.98	Black	Mesoamerican	1	Breeding line
KNG398	45.6	74.8	869.4	61.7	27.7	53.1	1.9	0.98	Black	Mesoamerican	3	Breeding line
KNG470	43.2	74.1	748.4	64.6	33.8	52.6	1.9	0.93	Black	Mesoamerican	1	Breeding line
KNG204	45.7	75.6	938.1	65	31.3	76.9	1.9	0.87	Black	Mesoamerican	1	Breeding line
KNG448	43.8	75	1009	64.8	30.6	62.9	1.9	0.85	Black	Mesoamerican	1	Breeding line
KNG494	42.2	74.4	809.2	70.4	33.6	53.2	1.9	0.73	Black	Mesoamerican	1	Breeding line
KNG474	41.4	75	857.9	64.7	33.2	53.4	1.9	-0.1	Black	Mesoamerican	2	Breeding line
KNG14	42.6	75.2	859.8	59.4	28.8	44.2	1.9	-0.3	Black	Mesoamerican	2	Breeding line
KNG179	45.7	76.6	663.8	66.8	31	52.4	1.9	-0.31	Black	Mesoamerican	1	Breeding line
KNG172	45.3	76.7	660.8	68.9	31.5	46.3	1.9	-0.37	Black	Mesoamerican	1	Breeding line
KNG423	44.1	78	820	75.5	37.2	70.5	2	-0.77	Black	Mesoamerican	1	Breeding line
ACC714	44.6	79.7	600.8	69.2	28.7	69.8	1.9	-3.24	Black	Mesoamerican	3	Variety
NCB226	42.8	73.7	914.9	60.9	28.8	52.3	1.9	0.96	Black	Mesoamerican	2	Breeding line
KNG27	40.8	75.6	817	61.2	29.4	50.9	1.8	-1.24	Others	Admixture	2	Breeding line
KNG24	39.2	74.8	780.8	63.4	31.4	50.1	1.8	-1.08	Others	Admixture	2	Breeding line
KNG39	43.9	76.8	704.8	66.5	30.5	52.9	1.8	-1.02	Others	Admixture	1	Breeding line
KNG281	43.2	76.2	604.4	79.6	33	67.6	1.9	-0.5	Others	Admixture	1	Breeding line
KNG409	44.3	76.9	790.7	75.4	34.5	52.4	1.9	-0.09	Others	Mesoamerican	1	Breeding line
KNG443	42.7	75.8	1058.8	70.8	31.1	68.5	2	0.26	Others	Mesoamerican	1	Breeding line
KNG26	43	74.5	729.3	66.1	30	50.7	1.9	0.31	Others	Mesoamerican	1	Breeding line
KNG202	44.9	76.3	849.7	73.4	36.8	38.2	1.9	0.89	Others	Mesoamerican	1	Breeding line
KNG482	43	74.2	968.3	65.1	30.2	49.6	2	1.1	Others	Mesoamerican	1	Breeding line
Averages												
Grand	44.1	75.2	791.0	64.2	28.9	73.0	1.9					
	41.3	73.6	709.1	63.3	27.8	71.5	1.9			Andean		
	44.9	75.7	825.8	63.9	28.9	73.6	1.9			Mesoamerican		
	43.1	75.4	672.6	67.3	30.9	72.1	1.9			Admixture		
	42.0	73.4	678.0	64.0	27.6	71.3	1.9		Yellow			
	41.3	73.6	730.8	62.6	28.0	72.4	1.9		Sugar			
	43.8	77.0	716.1	64.6	30.3	64.0	1.9		Large white			
	45.8	77.4	798.3	66.6	28.8	70.4	1.9		Small white			
	44.6	75.4	860.5	66.6	30.2	78.8	1.9		Small red			
	41.5	74.4	729.4	64.9	28.3	63.2	1.9		Red mottled			
	43.2	74.8	885.0	63.6	28.5	74.5	1.9		Red			
	40.8	73.2	657.6	66.9	29.3	62.8	1.9		Purple			
	45.4	75.7	815.8	61.8	27.7	76.1	1.9		Pinto			
	42.6	73.5	694.4	63.3	27.4	72.0	1.9		Cream (Mulatinho)			
	43.2	75.0	683.7	65.6	30.7	73.1	1.9		Brown			
	43.8	74.8	815.2	66.5	31.5	65.0	1.9		Black			

^a^Days to 50% flowering.

^b^Days to 50% maturity.

^c^Yield estimated in kg/ha.

^d^Seed iron concentration (ppm).

^e^Seed iron concentration (ppm)

^f^Cooking time (min).

^g^Hydration coefficient.

The average yield performance was 791 kg/ha, with 268 genotypes that yielded above the mean, and a variation of 397 to 1299 kg/ha (Fig 1c, Table 3). Most of the small and large red beans were among the best in YDHA while brown and purple were mostly poor (Fig 2). YDHA had no correlation to DPM and a positive but weak one (0.12) to DF (Fig 3). It had negative correlations of -0.12 and -0.18 to FESEED and ZNSEED, respectively (Fig 3). Seventy-one genotypes yielded better than all yield checks (released varieties; e.g., NABE 15, JESCA, MOORE 8802, Awash Melka, CAL 96, RWR 2154), which indicate a high potential for yield improvement (Fig 4 and Table 4). Several of these combined yield superiority to other traits (Fig 5). However, 89% of the 71 genotypes were Mesoamerican of various market classes, and only 8% were Andean of red mottled, sugar (speckled), and large red beans. The admixtures were all small red beans (Table 4). Mesoamerican beans had the highest yield average of 826 kg/ha compared to 709 kg/ha and 673 kg/ha obtained in the Andean and Admixed beans respectively (Table 4). The genotype BFS 10 was the most superior in yield (1299 kg/ha) (Table 4). Of the popular market classes in East Africa, very superior yielding yellow and large white beans were lacking in the population.

#### Cooking time

The hydration coefficient ranged from 1.6 to 2.1, with a mean of 1.9, which showed high soaking ability since 132 genotypes doubled (≥2.0) in weight after hydration (Fig 1d). All the market classes expressed superiority in soaking ability except small red genotypes (Fig 2d). The wide variability of 36 to 361 minutes showed diversity for cooking time (Fig 1e, Table 3), with most genotypes (321) cooked in less time than the mean value of 73 minutes. All market classes had fast and late cooking genotypes except small white beans which were all cooked in < 100 minutes (Fig 2). COOKT had weak and negative correlations to all traits except DF (Fig 3). A total of 42 genotypes cooked faster than the fast-cooking check variety, Awash Melka, which was cooked in 53 minutes (Fig 4 and Table 4). This was a substantial number of fast cooking beans, but most were Mesoamerican (62%), followed by Andean and admixtures at 29% and 10%, respectively. They were red mottled, sugar (speckled), purple, and cream coloured beans (Table 4). There was no yellow or large white bean represented among the lines that cooked faster than the check. The admixtures were purple, white, and other coloured beans.

#### Iron (FESEED) and zinc (ZNSEED) concentration

FESEED ranged from 52 to 87 ppm (Fig 1f). The average was 64 ppm, and 244 genotypes had higher FESEED than average. The market classes which had more than one genotype with above 75 ppm of Fe were small red, small white and others (Fig 2f). A strong positive correlation of 0.76 existed between FESEED and ZNSEED (Fig 3). The high Fe check, RWR2154 (65 ppm), performed just slightly better than average. Only one genotype (KNG492) had 20 ppm more FESEED than the check. KNG492 is a small red admixture bean (Table 4). The best performing genotypes and those selected by optimized indices are shown in Figs 4 and 5. Of the genotypes better than the check, 70, 16 and 13% were Mesoamerican, Andean, and admixtures, respectively. More than 50% of the Mesoamerican beans were small white and red beans, black, cream, brown, yellow and others. Most Andean beans were red mottled, sugar (speckled), purple, cream, sugar and yellow. The admixtures were cream, purple, small red, sugar, white and others (Table 4).

ZNSEED ranged from 23 to 38 ppm (Fig 1g). The average was 29 ppm, and 184 genotypes had higher ZNSEED than the average. The black and unclassified (other) beans had genotypes that expressed superiority in ZNSEED (Fig 2g). The best performing genotypes and those selected by optimized indices are shown in Figs 4 and 5. The check, RWR2154 (27 ppm), performed below the average. Only one genotype (KNG485), had >10 ppm of ZNSEED above the check. KNG485 is a small red Mesoamerican bean (Table 4). The genotypes better than the check were mostly Mesoamerican (72%), followed by Andean (17%) and admixtures (11%). Mesoamerican beans were small red and white beans, black, cream, yellow, brown, and other colours (Table 4). The Andean beans were sugar (speckled), red mottled, cream, purple and yellow among others. All admixtures were cream, small red, brown, white, sugar (speckled) and others (Table 4).

### Population structure of the genotypes

**Fig 6:**
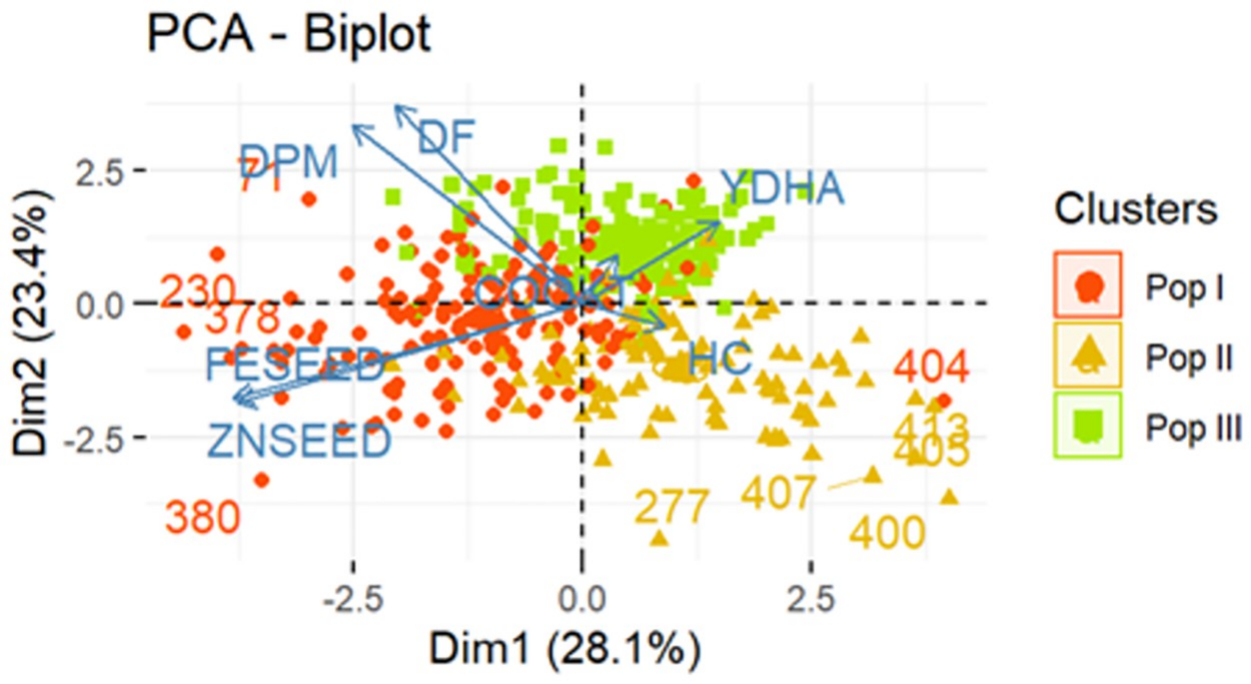
Biplot of 427 individuals and variables from principal component analysis, showing clusters predetermined by hierarchical clustering.

Phenotype-based cluster analysis separated the genotypes into three groups. These groups were illustrated in the PCA bioplot (Fig 6). Subpopulation I (Pop I) was 82% Mesoamerican, 8% Andean and 10% admixtures (Table 5).

**Table 5:** Mean performance (Best Linear Unbiased Prediction (BLUP)) and percentage of genotypes in the different gene pools for each of the three phenotype based population clusters.

Mean	DF^a^	DPM^b^	YDHA^c^	FESEED^d^	ZNSEED^e^	COOKT^f^	HC^g^	Andean	Mesoamerican	Admixture
								Percentage of genotypes		
Grand	44.1	75.2	790.9	64.1	28.9	73.0	1.9	20%	71%	8%
Population 1	44.4	75.4	788.7	66.7	30.5	75.3	1.9	10%	82%	0.08
Population 2	42.0	74.1	779.2	63.0	28.3	69.3	1.9	68%	20%	12%
Population 3	45.2	75.8	801.6	61.8	27.4	73.2	1.9	0%	98%	2%

^a^Days to 50% flowering.

^b^Days to 50% maturity.

^c^Yield estimated in kg/ha.

^d^Seed iron concentration (ppm).

^e^Seed iron concentration (ppm)

^f^Cooking time (min).

^g^Hydration coefficient.

Subpopulation II (Pop II) was predominantly Andean (68%), but with 20% Mesoamerican beans and 12% admixtures (Table 5). Subpopulation III (Pop III) was 98% Mesoamerican and 2% admixtures (Table 5). Pop I was the best in FESEED and ZNSEED based on means of 66.7 and 30.5 ppm, respectively. However, the group’s mean COOKT (75 min) was the highest and YDHA (788.7 kg/ha) the lowest (Table 5). Pop II recorded the lowest mean for COOKT (69.3 min), DF and DPM, and intermediate YDHA, FESEED and ZNSEED. Pop III had the highest mean YDHA (801.6 kg/ha) and an intermediate cooking time but recorded the lowest FESEED and ZNSEED (Table 5). The PCA revealed that the first three PC with eigen values of >1.0 accounted for 67.7% of the total variation among genotypes with the first two PC explaining 28.1% and 23.4% of the variations (Table 6). The major contributors for the first three PC were FESEED and ZNSEED for PC1, DF and DPM for PC2, and COOKT and HC for PC3 (Table 6).

**Table 6:** Eigen values and the principal component roots for 427 genotypes evaluated in different environments.

Attributes	PC^a^1	PC2	PC3	PC4	PC5	PC6	PC7
Eigenvalue	2.0	1.6	1.1	0.9	0.8	0.3	0.2
Variance (%)	28.1	23.4	16.2	13.3	11.5	4.1	3.4
Cumulative variance (%)	28.1	51.6	67.7	81.0	92.5	96.6	100.0
DF^b^	-0.44	0.80	-0.10	0.07	-0.05	0.37	0.06
DPM^c^	-0.54	0.73	-0.14	0.08	0.12	-0.37	-0.07
YDHA^d^	0.32	0.33	-0.04	-0.78	-0.41	-0.05	0.00
FESEED^e^	-0.82	-0.38	0.00	-0.10	-0.22	-0.06	0.34
ZNSEED^f^	-0.81	-0.40	0.03	-0.17	-0.16	0.08	-0.34
COOKT^g^	0.08	0.20	0.75	0.35	-0.52	-0.05	-0.02
HC^h^	0.19	-0.09	-0.73	0.37	-0.52	-0.03	-0.04

^a^Principal components.

^b^Days to 50% flowering.

^c^Days to 50% physiological maturity.

^d^Yield estimated in kg/ha.

^e^Seed iron concentration (ppm).

^f^Seed zinc concentration (ppm).

^g^Cooking time (min).

^h^Hydration coefficient.

The PCA biplot showed positive association of FESEED to ZNSEED, and DF to DPM (Fig 6). However, FESEED and ZNSEED were negatively associated to YDHA, as they were almost perfect opposites (Fig 6). COOKT and HC were the least contributors to variation. Genotypes in subpopulation I appeared in all quadrants but predominantly on the lower left, while those in subpopulation II were mostly in the lower right quadrant and those in subpopulation III were mostly in the upper right and left quadrants (Fig 6).

## Discussion

### The diversity of the genotypes

The significant differences among the evaluated genotypes indicated diversity in cooking time, FESEED, and yield, which is beneficial for crop improvement. The frequencies displayed many genotypes that performed better than average, which generally revealed the availability of many potential genotypes for breeding purposes. However, considering specific breeding targets, adding high Fe dense genotypes to the breeding population is recommended. Based on sufficient genetic variability that existed in a thousand accessions in the cultivated core collection of common bean in South America, breeding targets up to 80% and 50% potential improvement in Fe and Zn content above the baselines of 55 ppm of Fe and 35 ppm of Zn, respectively [35]. Subsequently, HarvestPlus set a threshold for high Fe beans (HIB) at 94 ppm, which has been realized through breeding in a few market classes such as white and red mottled [9, 36]. However, genotype x environment interaction is reported in FESEED [11, 12]. The results of this study also documented strong environment and GxE effects on FESEED, which accounted for more than 63% of the variance in FESEED. Such effects appear to make the high FESEED approach challenging for biofortification from a nutritional perspective. Defining the target population of environments such as sets of farms in which the varieties produced by a breeding program would be grown could lessen GxE effects on Fe thereby strengthening the nutrition perspective. In addition, a check strategy to assess genetic progress during breeding is essential. As such, an increment of 20 ppm of FESEED above the old variety to be replaced is expected in the HIB. In this study, only one genotype had 20 ppm more FESEED than the high Fe check, RWR2154. The mean concentration of 64 ppm for FESEED was low compared to other populations described in literature. The mean FESEED has increased from 65 ppm in the first regional nutrition nursery for East African bush beans [37] to 75–78 ppm in some recently improved varieties [10, 36, 38]. The low mean FESEED in this study is attributed to the nature of the population that was assembled for multiple traits. Nonetheless, the genotypes reported to concentrate ≥ 90 ppm of FESEED in the above-mentioned studies are potential candidates to increase diversity. A high diversity, and probably the use of modern breeding techniques are essential to attain breeding targets.

A key area of consideration is the bioavailability of Fe. The high Fe approach to bean FESEED biofortification assumes that bioavailability is maintained in most HIB. The association of high Fe to bioavailability is not yet very conclusive. Manteca (yellow) seed class was reported to combine high Fe, high Zn, and high bioavailable Fe [12]. Other recent studies indicate that Fe bioavailability is highly related to market class, essentially the polyphenolic profile of the seed coats [15, 16]. This indicates that polyphenols, and not phytate, are the dominant factor influencing Fe bioavailability. The yellow beans were reported to exhibit seed coat polyphenolic profiles that enhanced Fe bioavailability, but most market classes had inhibitory polyphenols [15, 16]. Previously, phytate was considered as the main factor influencing bioavailable Fe [13]. As it is positively correlated to concentration of bean FESEED, a reduction in phytate was proposed as an approach for high Fe beans [7, 13]. A low molar ratio of phytate to Fe implies a higher fractional absorption of Fe. However, in beans phytic acid levels are consistently in high molar excess relative to Fe, usually at a 10:1 or higher ratio, thus the inhibitory effect is near or at maximal [14]. So, the breeding approach would probably necessitate modern breeding technologies such as genome editing or mutation. In a study of beans in the East African marketplace, Fe concentration and bioavailability measurement indicated the biofortified bean varieties are providing no additional dietary Fe [39]. Nonetheless, most of the studied genotypes that were identified as “biofortified” were not improved varieties, but old varieties released as HIB from the baseline study. A study including more biofortified lines is recommended to confirm these findings. It is essential to keep in mind that increases in Fe concentration would be meaningless if the Fe is not bioavailable, unless dietary recommendations include enhancers like ascorbic acid. The influence of GxE on bioavailable Fe was reported minimal in study of 12 genotypes in two field seasons across nine on-farm locations in three agro-ecological zones in Uganda where 68.3% of the phenotypic variation was attributed to the genotypes [12].

Most of the genotypes were cooked in a shorter time than the population mean, and up to 42 attained a shorter cooking time than the fast-cooking check, Awash Melka. All market classes had at least one fast-cooking genotype, but the small white beans were notably outstanding since all genotypes were cooked in less than 100 minutes. The type is widely preferred in some places because of their fast-cooking ability [40]. The availability of fast cooking beans in all market classes is an advantage for trait improvement within the preferred market class. While it took 36 mins for the fastest cooking genotype in this study to cook, other studies reported genotypes that were cooked in 14 to 29 minutes using the same method [38, 41, 42]. Variations could be due to genetics or conditions during seed production or storage. The fast-cooking beans reported in the above-mentioned studies are potential candidates for increasing variation in this population. A further investigation to understand the factors responsible for the earliness to cook in the different market classes is recommended to inform breeding decisions.

A mean hydration coefficient of 1.9 showed a good soaking ability for the population, where 132 genotypes increased at least twice in mass. The small red bean market class generally reflected the poorest soaking ability even though some hydrated well and cooked fast. Similar soaking ability like observed in this study was previously reported [10, 42]. The high swelling capacity of beans was largely a trait for the canning industry that is used to reflect a higher can weight [43]. It is gaining importance as a consumer trait since it reflects more food quantity on the plate. The observed variation reflected a high potential for improvement of this trait. A further study on the cause of variation in the soaking ability within the small red market class, and beans from other market classes that poorly soaked, could provide more understanding on fast cooking ability.

### Population structure of the genotypes

The three clusters generated did not reveal a clear separation of genotypes based on the similarity in mean performance of the groups and spread by the first two PC. This clumping of genotypes showed the possibility for multiple trait selection. Lines that expressed superiority in some traits but performed poorly in others also existed. Hybridization of elite x elite parent are important for demand-led breeding since repulsion linkages can be broken up over time, resulting in substantial variation in most important economic traits. However, maintaining long-term viability necessitates regular addition of new diversity in elite breeding populations [19]. In consideration of this, improvement strategy could focus on each group separately. Improvement targeting high FESEED and ZNSEED with moderate YDHA for subpopulation I, fast cooking beans with moderate FESEED, ZNSEED and YIELD for subpopulation II and high YDHA with moderate COOKT for subpopulation III. This is because the negative association of FESEED and ZNSEED to YDHA may not allow a single population improvement strategy without any penalties. Thirteen co-located quantitative trait loci (QTL) that would negatively influence yield components and FESEED and ZNSEED were reported, but several QTL for FESEED (eight) and ZNSEED (six) also segregated independently of yield components, which showed the possibility of improving the three traits concurrently [44]. For COOKT and YDHA, the weak correlation [r = -0.01] showed unlikely yield penalty during breeding for fast cooking beans if careful breeding and selection decisions are made.

Considering gene pools, subpopulations I and II were predominantly Mesoamerican and Andean, respectively, but with some Andean and admixtures. Most of the subpopulation III lines were Mesoamerican but with some admixtures. The subpopulations that were predominantly Mesoamerican (788.7 and 801.6 kg/ha) yielded better than Andean (779.2 kg/ha). Therefore, yield improvement plans could carefully consider inter-gene pool crosses while incorporating the weight of 100 seeds in the selection index.

## Conclusions

The red, black, and cream market classes had the highest genetic diversity. The observed variations within and among subpopulations were insightful to guide product profile development to realize genetic gain. Improvement within the market classes in Mesoamerican gene pool is feasible for YDHA, COOKT, FESEED, ZNSEED especially for small red, small white and black beans, due to the presence of many superior genotypes. Similarly, the Andean red mottled, sugar (speckled), large red and purple beans could be crossed within the market classes. However, significant improvement in FESEED may require other sources of high Fe beans or more elaborate crossing schemes. Further evaluation for traits related to bioavailable Fe is essential. Although there was genetic diversity in yellow beans, superiority in phenotypic traits was uncommon; improvement plans could consider other sources of yellow beans or crossing with close colours such as cream or brown. A further investigation to understand the factors responsible for the earliness to cook in the different market classes is recommended to inform breeding decisions. Understanding the cause of high variation in the soaking ability within the small red market class, and other beans from other market classes that poorly soaked, could provide more insights on fast cooking ability.

## Supporting information

S1 File (CSV)

S2 File (CSV)
